# Real-life follow-up after discontinuation of anti-PD1 therapy for complete-response cutaneous squamous cell carcinoma in patients over 75 years of age

**DOI:** 10.1093/oncolo/oyaf382

**Published:** 2025-11-17

**Authors:** Julie Lardenois, Eve Desmedt, Sophie Maiezza, Marie Bridoux, Laurent Mortier, Marie Boileau

**Affiliations:** Department of Dermatology, Univ. Lille, CHU de Lille, Lille 59037, France; Department of Dermatology, Univ. Lille, CHU de Lille, Lille 59037, France; Department of Dermatology, Univ. Lille, CHU de Lille, Lille 59037, France; Departement of Oncology, Univ. Lille, CHU de Lille, Lille 59037, France; Department of Dermatology, Univ. Lille, CHU de Lille, Lille 59037, France; U1189-ONCO-THAI-Assisted Laser Therapy and Immunotherapy for Oncology, Inserm Laboratory, Inserm, Univ. Lille, Inserm, CHU de Lille, Lille 59037, France; Department of Dermatology, Univ. Lille, CHU de Lille, Lille 59037, France; U1189-ONCO-THAI-Assisted Laser Therapy and Immunotherapy for Oncology, Inserm Laboratory, Inserm, Univ. Lille, Inserm, CHU de Lille, Lille 59037, France

**Keywords:** checkpoint inhibitors, immunotherapy, elderly, cutaneous squamous cell carcinoma, discontinuation, anti-PD1, real-life

## Abstract

**Introduction:**

Metastatic or locally advanced cutaneous squamous cell carcinomas (CSCC) affect mainly individuals over 75 years of age. Anti-PD1 therapy is the first-line treatment. Limited data are available on the continuation of immunotherapy after complete response (CR) has been achieved.

**Materials and methods:**

We present a retrospective monocentric study of patients over 75 years of age treated with anti-PD1 therapy for CSCC after early immunotherapy discontinuation. Patients were treated between 01/2019 and 01/2024.

**Results:**

We identified 44 patients over 75 years of age treated with anti-PD1 therapy, 14 of whom achieved CR, leading to the discontinuation (31%). Median age was 83.5 years. Tumor were located on the head and neck in 92.9% of cases. Median follow-up was 20 months. Median time to CR was 5 months. Discontinuation occurred 1.3 months after the CR diagnosis. One-third of the patients experienced no adverse events. Only one patient experienced grade 3 toxicity, and 10 months after discontinuation of treatment, experienced a nodal recurrence. One death occurred 8 months after CR and was unrelated to CSCC or treatment.

**Conclusion:**

Anti-PD1 therapy is effective and safe in elderly patients. CR can be achieved quickly and maintained, despite early treatment discontinuation. The use of anti-PD1 therapy is therefore encouraged even in this fragile and comorbid population, with discontinuation planned as soon as a complete response is obtained to limit the duration of treatment and promote quality of life for patients. Further studies and prolonged follow-up are needed to establish guidelines regarding anti-PD1 discontinuation.

Implications for Practice

- Real-life study of elderly patients (>75 years) with advanced cutaneous squamous cell carcinoma treated with anti-PD1
- Thirty-one percent of patients achieved complete response allowing early treatment discontinuation
- Median time to complete response was 5 months (6 infusions)
- Excellent safety profile with minimal toxicity after early discontinuation (1.5 months after complete response).
- Durable responses support early anti-PD1 withdrawal in frail elderly patients

## Introduction

Cutaneous squamous cell carcinoma (CSCC) is the second most common type of skin cancer.^1^ It affects mainly elderly individuals. The median age at diagnosis is approximately 74 years in men and 77 years in women.^2^ Three percent of CSCC cases progress to lymph node involvement or metastasis. The death rate varies from 1% to 3% depending on the study.^3^ The incidence has increased by 50%-200% annually over the last three decades, mainly due to an aging population and changes in tanning habits.^4^ Elderly subjects represent a heterogeneous population,^5^ often comorbid, for whom the aim of the treatment is often to relieve symptoms. In France, individuals 75 years of age and older are admitted to a geriatric department. The population is aging, and those over 75 represent 10.4% of the French population as of 2024, compared with 9% in 2013.^6^ Anti-PD1 agents have proven effective against locally advanced or metastatic CSCC.^7–9^ Cemiplimab has been approved for first-line treatment for this condition since June 2019, and its cost has been a covered by healthcare expenses in France since October 2024 as a second-line treatment or as a treatment for patients with contraindications to chemotherapies. However, we have few data on the continuation of immunotherapy after a complete response in patients with CSCC. Anti-PD1 drugs are usually discontinued after 6 months to a year of CR, similar to what is done in melanoma treatment.

We aimed to investigate the feasibility of discontinuing immunotherapy early in vulnerable, elderly patients who may benefit from treatment in terms of quality of life but for whom the prolonged continuation of immunotherapy could be harmful. The present study proposes to evaluate the rate of recurrence after early discontinuation of immunotherapy in this vulnerable population.

## Methods

We conducted a monocentric retrospective study at Lille University Hospital. Patients were identified via the use of chemotherapy prescribing software. We identified patients over 75 years of age treated with anti-PD1 therapy for locally advanced or metastatic CSCC. Patients were treated between January 2019 and January 2024. Data were collected until May 2024. Complete response was assessed clinically via computed tomography (CT) or positron emission tomography (PET) scans. The data collected included sex, age, Eastern Cooperative Oncology Group (ECOG) performance status, number of treatments, notable patient history, primary location of cutaneous squamous cell carcinoma, carcinoma stage, and the PDL1 combined positive score (CPS), which represents the number of PDL1-stained cells (tumor cells, lymphocytes, and macrophages) divided by the total number of viable tumor cells multiplied by 100. We also collected data on previous treatments, the type of anti-PD1 agent used, the presence or absence of associated radiotherapy, and the reason for stopping immunotherapy. We evaluated the time to complete response (CR), duration of immunotherapy continuation after CR, progression-free survival (PFS) and overall survival (OS) after immunotherapy discontinuation, median total follow-up (from the start of immunotherapy to the date of last news), recurrence after immunotherapy discontinuation, death rate during follow-up and adverse effects. Response to treatment was assessed by an independent radiologist using RECIST 1.1 for imaging, or investigator assessment of clinical response. The results are expressed with medians and interquartile ranges (Q1-Q3). The work has been declared to the CNIL (DEC24-241).

## Results

**Figure 1.**
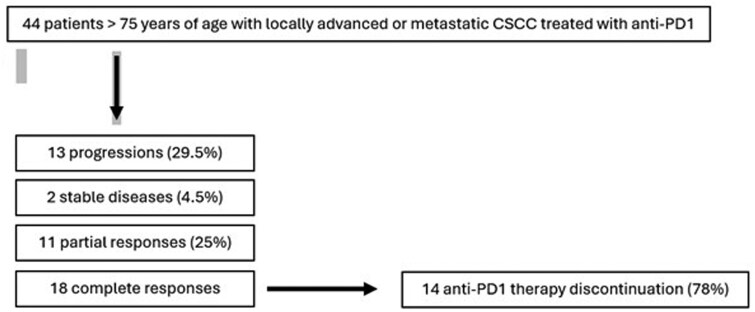
Flow chart.

We identified 44 patients over 75 years of age treated with anti-PD1 therapy for CSCC in our department between January 2019 and January 2024. Among them, there were 13 progressing patients (29.5%), 2 stable patients (4.5%), 11 partial response patients (25%) and 18 patients with a CR (41%). Among these 18 patients, treatment was discontinued for 14 (78%). These data are presented in Figure 1.

The objective of this study is to examine the outcomes of immunotherapy in a cohort of 14 patients who exhibited a complete response and subsequently had the treatment discontinued. The patients’ characteristics are presented in Table 1. The majority were men (78.6%). The median age was 83.5 years, with a minimum of 75 years and a maximum of 92 years. Most patients were highly comorbid, with 85.7% of patients having a cardiovascular history of hypertension, myocardial infarction, rhythm disorders, heart failure or type 2 diabetes. Nearly one-third of the patients were diagnosed with neurological pathologies (dementia, Parkinson’s disease, etc). A history of other skin cancers was reported by 42.9% of patients, and 2 patients had previously been treated for noncutaneous cancer. None of the patients were immunocompromised. Two patients had autoimmune comorbidities. The median number of medications was 6 (Q1 4-Q3 8). The ECOG performance status was 1 for 42.9% of patients, 2 for half, and 3 for one patient (7.1%). An oncogeriatric evaluation was carried out for three patients. For each of these 3 patients, the evaluation was in favor of immunotherapy.

**Table 1.** Population characteristics.

Categories		*N* = 14	%
**Gender**	Women	3	21.4
	Men	11	78.6
**Age**	Median [Q1-Q3]		83.5 [80.5-86.8]
**ECOG Performance Status**	0	0	0
	1	6	42.1
	≥2	8	57.1
**Number of treatments**	Median [Q1-Q3]		6 [4.25-7.75]
**Comorbidities**	Immunodepression	0	0
	Autoimmune (thyroiditis, type 1 diabetes, lupus, etc.)	2	14.3
	Cardiovascular (hypertension, heart attack, rhythm disorders, heart failure, type 2 diabetes, etc.)	12	85.7
	Neurological (MS, dementia, etc.)	4	28.6
	Other skin cancers	6	42.9
	Other cancers	2	14.3
**Primitive site**	Head and neck	13	92.9
	Trunk	1	7,1
**Stage**	Locally advanced	8	57.1
	Lymph node involvement	4	28.6
	Distant metastases	2	14
**Primary lesion or recurrence**	Primary lesion	6	42.9
	Recurrence	8	57.1
**CPS**	Unknown	7	50
	≥ 1	7	100
	≥ 20	3	42.9
**Previous treatments**	Surgery	9	64.3
	Chemotherapy	0	0
	Radiotherapy	2	14.3
	Brachytherapy	1	7.1
**Molecule**	Pembrolizumab	9	64.3
	Nivolumab	3	21.4
	Cemiplimab	2	14.3
**Associated radiotherapy**	No	9	64.3
	Initial	3	21.4
	Closing	2	14.3
**Cause immunotherapy discontinuation**	RCP choice for complete response	13	92.9
	Toxicity	1	7.1

The head and neck were the primary tumor sites in 92.9% of patients and the trunk in one patient. Patients were treated for primary lesions in 42.9% of cases and for recurrence after surgery, radiotherapy or brachytherapy in 57.1% of cases. CSCCs were locally advanced in 57.1% of patients, with lymph node involvement in 28.6% and distant metastasis in 14%. The PDL 1 CPS was assessed in 50% of patients; it was positive (ie, ≥ 1) in 100% of patients tested and had a value of over 20 in 42.9% of patients tested. Radiotherapy or brachytherapy had been previously performed in three patients.

**Figure 2.**
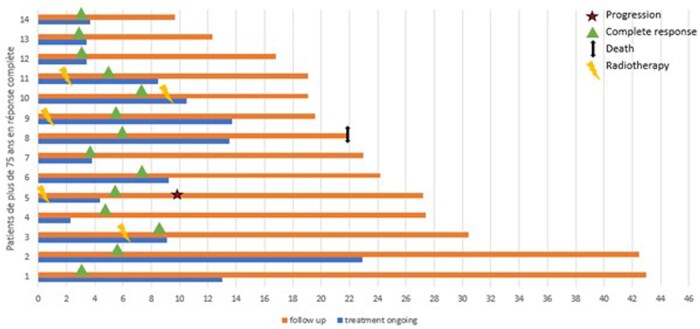
Time to complete response and duration of complete response in elderly patients treated with anti-PD1 therapy for locally advanced or metastatic CSCC (months).

Anti-PD1 therapy was the first-line systemic treatment for all patients. Pembrolizumab was used in 64.3% of patients, nivolumab in 21.4%, and cemiplimab in 14.3%. In association with immunotherapy, initial radiotherapy was carried out in three patients, and closure radiotherapy was performed on two patients. Immunotherapy was discontinued after multidisciplinary tumor board decisions were made for all patients. In 92.9% of patients, this was due to their general condition and advanced age. In one patient (patient 5), treatment was discontinued after diagnosis of grade 3 pulmonary toxicity concomitant with the diagnosis of CR. Figure 2 shows the results of our follow-up on these patients. The results are reported in Table 2. The median total patient follow-up period was 20 months (Q1 17.1-Q3 25.5). The median time to CR was 5.1 months (Q1 3.3-Q3 5.9). The median number of infusions was 12 (Q1, 6-Q3, 15, 75). The median duration of treatment after CR was 1.3 months (Q1 0.5-Q3 6.5). The median overall survival after terminating immunotherapy was 14.2 months (Q1 8.7-Q3 20.8). Only one patient experienced inguinal nodal recurrence 8,6 months after discontinuation of the treatment. It is important to note that this patient was the sole case of trunk localization, and immunotherapy had to be discontinued due to toxicity occurring at the same time of the diagnosis of CR (patient 5). The patient was treated with a reintroduction of immunotherapy and radiotherapy.

**Table 2.** Results.

	*N* = 14	[Q1-Q3] or %
**Median progression-free survival after immunotherapy discontinuation [Q1-Q3]**	8.6 months	[5.9-13.7]
**Median overall survival after immunotherapy discontinuation [Q1-Q3]**	14.2 months	[8.7-20.8]
**Median time to complete response [Q1 Q3]**	5.1 months	[3.3-5.9]
**Median duration of treatment after complete response [Q1-Q3]**	1.3 months	[0.5-6.5]
**Median total follow-up [Q1-Q3]**	20 months	[17.1-25.5]
**Progression after immunotherapy discontinuation**	1	7.1%
**Deaths**	1	7.1%

**Figure 3.**
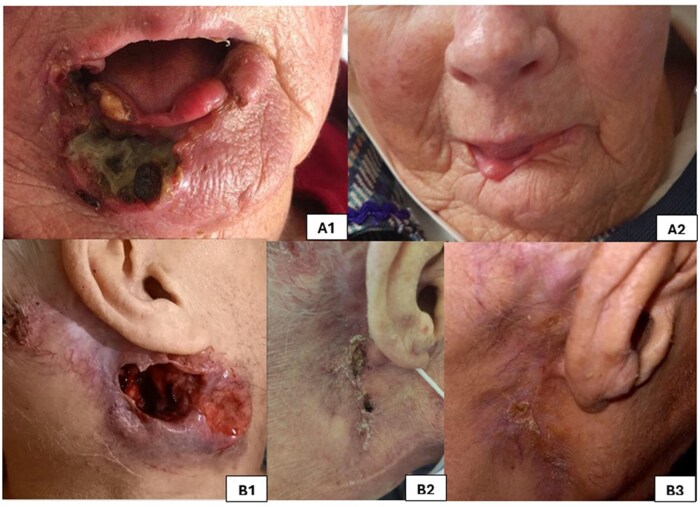
Illustrations of two patients. (A) Patient number 7. A1 before treatment. A2 Three months after discontinuation—five infusions of pembrolizumab. (B) Patient number 4. B1 Before treatment. B2 After three infusions of pembrolizumab. B3 Three months after discontinuation—four infusions of pembrolizumab.

Finally, one patient died during follow-up, and the death was unrelated to CSCC or to treatment. Two patient illustrations are shown in Figure 3.

Complete response was assessed clinically for all the patient and by imaging in 78.6% of patients (18% by PET, 18% by MRI and 63.6% by CT). Indeed, assessment of complete response was performed only clinically for 2 altered patients with locally advanced cutaneous epidermoid carcinoma

Safety data are reported in Table 3. More than one-third of the patients did not experience any toxicity (35.7%). Grade 1 toxicity was reported in 35.7% of patients: the most common symptom was fatigue (5 patients), followed by pruritus (1 patient), eczema (1 patient), hyperthyroidism (1 patient), and hypereosinophilia (1 patient). Grade 2 toxicities were present in four patients (28.6%): two patients had adrenal insufficiency, one patient had lymphopenia, and one patient had neutropenia. Only one patient experienced grade 3 toxicity, manifesting as diffuse interstitial pulmonary disease. This patient was the sole case of recurrence following discontinuation, and this case of lymph node recurrence was treated with radiotherapy.

**Table 3.** Safety data.

Toxicity			*N* = 14	%
**Grade 1**	Number of patients		7	50
	Type of toxicity	Asthenia	5	35.7
		Pruritus	1	7.1
		Eczema	1	7.1
		Hyperthyroidism	1	7.1
		Hypereosinopilia	1	7.1
**Grade 2**	Number of patients		4	28.6
	Type of toxicity	Pruritus	1	7.1
		Adrenal insufficiency	2	14.3
		Lymphopenia	1	7.1
		Neutropenia	1	7.1
**Grade 3**	Number of patients		1	7.1
	Type of toxicity	Diffuse interstitial lung disease	1	7.1

## Discussion

In this real-life cohort, we confirmed that anti-PD1 therapy is effective and safe for the treatment of locally advanced or metastatic cutaneous squamous cell carcinomas in elderly patients over 75 years of age. Despite this early cessation, after discontinuation of treatment for complete response, only one patient experienced a relapse. This was the only patient for whom immunotherapy was stopped for grade ≥ 3 toxicity and the only patient for whom CSCC was localized outside of the head and neck. Only one death occurred, which was unrelated to treatment and CSCC. It occurred 8 months after stopping immunotherapy.

This elderly, comorbid population is often excluded from clinical trials because of an excessively long medical history, an Eastern Cooperative Oncology Group (ECOG) performance status ≥ 2 or difficulties in providing free and informed consent. Presley et al. reported that the efficacy and toxicity of immunotherapy in fit older adults and younger patients seem to be comparable.^10^

In the TOSCA study, the authors demonstrated the efficacy of cemiplimab against locally advanced or metastatic CSCC in patients with a median age of 81 years. The median PFS was 14 months, with a median follow-up of 20 months and a CR rate of 30%.^9^

In the EMPOWER-CSCC study evaluating cemiplimab, the CR rate was approximately 17%, with an overall median duration of response of 41.3 months.^12^ In the KEYNOTE-629 study evaluating pembrolizumab, the CR rate was 10.5% in patients with metastatic CSCC and 16.7% in those with locally advanced disease.^7^ A study by Samaran et al. evaluating the efficacy of anti-PD1 therapy in CSCCs in patients over 70 years of age revealed a CR in 19% of patients.^11^ The higher rate of CR in our cohort may be explained in part by a more often locally advanced disease with fewer distant metastases in our cohort and the fact that part of the complete response was established by PET scan with complete metabolic responses but partial radiological responses. Furthermore, most of the patients in pivotal studies had already received a first or second line of systemic treatment, whereas in our study, they were all naïve to systemic therapy. In the study by Samaran et al., 15% of the patients were immunosuppressed, whereas none in our study were immunosuppressed. In addition, CSCC were located on the head and neck in 77.8% of the patients. This enhanced response in head and neck CSCC could be explained by a greater tumor mutational burden in this chronically UV-exposed location. Rodrigo et al. described a better response to immunotherapy in head and neck SCC with a higher mutational load.^13^

In our experience, clinicians can predict whether a patient will benefit from treatment after 2 to 3 courses of treatment. Indeed, patients who present a complete response generally present a partial clinical response very quickly after the introduction of treatment. Notably, the time to CR in our patients was rapid, with a median time of 5.1 months (ie, approximately 6 courses of immunotherapy). The maximum time to CR was 8.6 months. These data concur with those of the study by Khelef et al., where the median time to CR was 6.1 months.^14^

Macaire et al. evaluated the continuation of immunotherapy afterprogression in advanced melanoma patients and reported no significant grade >3 adverse events in theprolonged treatment group [15]. Few studies have examined the discontinuation of immunotherapy in patients with CSCC. Rulz et al. reported no improvement in the CR rate when immunotherapy was continued beyond 12 months compared with 6-12 months.^16^ Nevertheless, there are currently no recommendations for the continuation of immunotherapy once a complete response has been achieved. CSCC affects mainly elderly individuals with comorbidities. The management of this vulnerable population, thus, gives rise to questions regarding the acceptability of treatment. The objective of the treatment is to provide symptomatic treatment for CSCC while minimizing the adverse effects and the inconvenience associated with coordinating treatment. In our study, quality of life also improved in these patients evaluated with patient-reported outcomes. Patient 4 showed decreased pain and was able to discontinue use of tier 3 analgesics (Figure 3), and Patient 7 was able to resume a normal diet.

The benefit of continuation of anti-PD1 therapy after a CR unclear and patients may experience late adverse effects. Indeed, Samaran’s study revealed a deterioration in the performance status of 41% of patients as well as in the nutritional status, with weight loss in 57% of patients, even in the absence of direct immunotherapy toxicity.^11^ Continuation of treatment in the absence of any contribution to disease control is debatable.

In addition, the cost of continuing immunotherapy is also questionable. Paul et al. revealed that the costs of treatment with cemiplimab and pembrolizumab for CSCC are $683 061 and $320 817, respectively. In France in 2024, the price of an injection of pembrolizumab 200 mg, cemiplimab 350 mg or nivolumab 480 mg was 5200 euros, 3318 euros, and 4860 euros, respectively. The early discontinuation of immunotherapy following a CR has the potential to reduce costs, which is a rational approach given the prevailing concern regarding the limitation of healthcare expenses.^17^

Immunotherapy is usually continued for 6 months to 1 year after a CR has been achieved, analogous to the treatment of melanoma. The study by Khelef et al. reported the experiences of 46 patients in whom anti-PD1 therapy was discontinued when a CR was achieved.^14^ The median time from CR to discontinuation of immunotherapy was 3.1 months. The median follow-up was 16.9 months. No recurrences were reported during this period.

In our study very early cessation of immunotherapy after complete response was done with the median delay of 1.3 months (cessation as soon as a complete response was observed). This delay can be attributed to the time required to obtain the results of external imaging in the local area and to organize the discussion of all cases in multidisciplinary tumor boards. Our results support the original limited-dose studies which showed extremely high CR rates. The current practice for the treatment of cancers, including CSCC, is to propose neoadjuvant treatment. Once a satisfactory clinical response has been obtained, most patients do not want further treatment. This was the case for approximately ten patients in the MATISSE trial.^18^ In this population, it seems difficult to take an operative risk when a complete response can be obtained with a few more infusions.

The study does, of course, have its limitations with a small sample size, a retrospective and single-center design, an heterogeneous CR assessment methods with a lack of standardized follow-up imaging, the absence of a comparator arm or surgical comparison group, and potential underreporting of toxicities due to the retrospective design. However, this was a real-life study involving unselected, comorbid and multimedicated patients. The follow-up period was prolonged compared to other studies, with a median time of 20 months. A quarter of patients had a follow-up period lasting for more than 2 years. The efficacy and safety of anti-PD1 therapy in elderly patients with locally advanced or metastatic CSCC has been demonstrated in this small retrospective cohort, with a persistent CR after the rapid discontinuation of anti-PD1 therapy.

It is imperative that prospective studies with extended follow-up periods are conducted to substantiate these observations and formulate evidence-based guidelines Furthermore, the utilization of predictive markers of response to anti-PD1 therapy for the selection of patients’ merits consideration.

## Conclusion

The use of anti-PD1 agents as a first-line treatment modality for locally advanced or metastatic cutaneous squamous cell carcinoma has been demonstrated to be efficacious. It appears that a complete response is sustained following the premature cessation of immunotherapy. This early discontinuation would have the effect of limiting mobilization and costs for this fragile population, with a concomitant benefit in terms of symptoms.

## Data Availability

The data underlying this article will be shared on reasonable request to the corresponding author.
